# Supplementary material for: Correlation of Taste Components with Consumer Preferences and Emotions in Chinese Mitten Crabs (Eriocheir sinensis): The Use of Artificial Neural Network Model
Source: Foods. 2022 Dec 19;11(24):4106. doi: 10.3390/foods11244106 (PMC9777666; doi:10.3390/foods11244106)
Supplement: Supplementary file 1 [file foods-11-04106-s001.zip › foods-2048995-supplementary.pdf]

Table S1

Basic information on the Chinese mitten crab

	**C-JH**	**T-JH**	**T-CJ**
Origin	Chongming	Taixing	Taixing
Variety	Jianghai 21	Jianghai 21	Yangtze II
Breeding (n=3)			
Type of feed	Mixed grains ,fish and Whelk	Mixed grains and fish	Mixed grains and fish
pH	8.38 ± 0.19^{a}	7.30 ± 0.20^{b}	6.72 ± 0.13^{c}
Salinity/%	0.10 ± 0.00^{b}	0.30 ± 0.00^{a}	0.30 ± 0.00^{a}
Temperature/℃	19.52 ± 0.23^{c}	20.66 ± 0.21^{b}	21.96 ± 0.09^{a}
Transparency/cm	36.30 ± 4.47^{c}	66.68 ± 5.58^{a}	51.72 ± 1.57^{b}
Yield (n=5)			
Weigh/g	136.10±10.57	137.80±13.81	124.11±8.6
Gsi/%	9.82 ± 0.36^{a}	8.67 ± 1.44^{ab}	7.72 ± 1.27^{b}
Hsi/%	7.08±0.70	7.85±0.56	7.90±0.61
MY/%	15.03±1.12	13.67±1.59	13.34±1.41
TEY/%	31.93 ± 0.79^{a}	30.20 ± 0.84^{b}	28.95 ± 1.06^{c}

Note: Gsi (gonadosomatic index), Hsi (hepatopancreas index), MY (meat yield), TEY (total edible yield); Chongming origin; T: Taixing origin; JH: Jianghai 21 variety; CJ: Yangtze II variety

Table S2

Basic information on the consumers (n = 93)

	Consumers
Age	23.1±1.7
Gender (% female)	60.22
District from (%respondents)	
Jiangsu province	16.13
Shanghai city	5.38
others	78.49
Frequency of using emoji (% respondents)	
Always	30.77
Often	43.59
Sometimes	12.82
Seldom	7.69
Never	5.13
Frequency of consumption of aquatic products (% respondents)	
Daily	2.56
Multiple times a week, but not daily	20.51
Weekly	41.03
Monthly	30.77
Never	5.13
